# Disparities in outcomes of colorectal cancer surgery among adults with intellectual and developmental disabilities

**DOI:** 10.1371/journal.pone.0308938

**Published:** 2024-08-27

**Authors:** Ayesha P. Ng, Shineui Kim, Nikhil Chervu, Zihan Gao, Saad Mallick, Peyman Benharash, Hanjoo Lee

**Affiliations:** 1 Cardiovascular Outcomes Research Laboratories, David Geffen School of Medicine at UCLA, Los Angeles, CA, United States of America; 2 Department of Surgery, Division of Cardiac Surgery, David Geffen School of Medicine at UCLA, Los Angeles, CA, United States of America; 3 Department of Surgery, Division of Colon and Rectal Surgery, Harbor-UCLA Medical Center, Torrance, CA, United States of America; Covenant University, NIGERIA

## Abstract

**Background:**

Disparities in colorectal cancer screening have been documented among people with intellectual and developmental disabilities (IDD). However, surgical outcomes in this population have yet to be studied. The present work aimed to evaluate the association of IDD with outcomes following colorectal cancer resection.

**Methods:**

All adults undergoing resection for colorectal cancer in the 2011–2020 National Inpatient Sample were identified. Multivariable linear and logistic regression models were developed to examine the association of IDD with risk factors as well as outcomes including mortality, complications, costs, length of stay (LOS), and non-home discharge. The study is limited by its retrospective nature and did not capture disease staging or time of diagnosis.

**Results:**

Among 722,736 patients undergoing colorectal cancer resection, 2,846 (0.39%) had IDD. Compared to patients without IDD, IDD patients were younger and had a higher burden of comorbidities. IDD status was associated with increased odds of non-elective admission (AOR 1.40 [95% CI 1.14–1.73]) and decreased odds of treatment at high-volume centers (AOR 0.64 [95% CI 0.51–0.81]). Furthermore, IDD patients experienced significantly greater LOS (9 vs 6 days, p<0.001) and hospitalization costs ($23,500 vs $19,800, p<0.001) relative to neurotypical patients. Upon risk adjustment, IDD was significantly associated with 2-fold increased odds of mortality (AOR 2.34 [95% CI 1.48–3.71]), 1.4-fold increase in complications (AOR 1.41 [95% CI 1.15–1.74]), and 6.8-fold increase in non-home discharge (AOR 6.83 [95% CI 5.46–8.56]).

**Conclusions:**

IDD patients undergoing colorectal cancer resection experience increased likelihood of non-elective admission, adverse clinical outcomes, and resource use. Our findings highlight the need for more accessible screening and patient-centered interventions to improve quality of surgical care for this at-risk population.

## Introduction

Intellectual and developmental disabilities affect over 3 million people in the United States and have been associated with increased risk for colorectal cancer [1–3]. Individuals with IDD face higher rates of physical and mental health issues, adverse social determinants of health including poverty and social isolation, and barriers to adequate communication [4–6]. The combination of both individual and structural-level inequities impedes access to optimal cancer care and often leads to late-stage diagnoses [7]. Screening for early detection of colorectal cancer contributes to improved survival but is underutilized among patients with IDD compared to the general population [8].

Surgical resection is the standard treatment for colorectal cancer, and disparities in colorectal surgical care have been well-documented. Differences in timely progression to surgery, operative approach, and complication rates following colorectal cancer resection have been reported based on race and ethnicity, socioeconomic status, and insurance status [9, 10]. However, literature on surgical care among the IDD population is scarce. A recent study on emergent general surgery demonstrated delayed presentation and inferior outcomes among IDD patients compared to neurotypical patients [11]. Another study on kidney transplantation reported lower rates of evaluation and transplant among individuals with IDD, suggesting systemic discrimination [12]. In the context of colorectal cancer surgery, studies on IDD are lacking and a deeper understanding of disparities in outcomes is warranted to improve quality of surgical care for this at-risk population.

The present work used a nationally representative cohort of patients undergoing resection for colorectal cancer to evaluate the association of IDD with patient, operative, and hospital characteristics. In addition, we analyzed in-hospital clinical and financial outcomes following resection. We hypothesized that patients with IDD would have significantly increased odds of mortality, complications, costs, and length of stay.

## Methods

This was a cross-sectional study using the 2011–2020 National Inpatient Sample (NIS). Maintained by the Healthcare Cost and Utilization Project (HCUP), the NIS is the largest publicly available all-payer inpatient database in the United States [13]. Using robust survey weighting algorithms, the NIS provides accurate estimates for approximately 97% of all US hospitalizations. Due to the de-identified nature of the NIS, this study was deemed exempt from full review by the Institutional Review Board at the University of California, Los Angeles (accessed January 18, 2024).

All adult patients (≥18 years) with a diagnosis of colon or rectal cancer undergoing colectomy (right, transverse, left, sigmoid, total) or rectal resection were identified using relevant *International Classification of Diseases 9th/10th Revision* (ICD-9/10) diagnosis and procedure codes (S1 Table). Patients with IDD were identified based on the presence of ICD-9/10 diagnosis codes for intellectual disability, pervasive developmental disorders, cerebral palsy, or Down syndrome (S1 Table). Patient and hospital characteristics including age, sex, race, income quartile, primary payer, admission type, and hospital region and teaching status were defined using the HCUP Data Dictionary [13]. Records with missing data for admission type, age, sex, race, income, payer, or mortality were excluded from further analysis.

Comorbidities such as diabetes, hypertension, obesity (defined as BMI > 30), coronary artery disease, chronic pulmonary disease, and chronic liver disease were identified using ICD-9/10 diagnosis codes (S1 Table). The Elixhauser Comorbidity Index, a validated composite of 30 comorbidities, was additionally used to quantify the overall burden of chronic conditions [14]. Presence of bowel obstruction, minimally invasive operative approaches (laparoscopic and robotic), and ostomy procedures were also captured using ICD-9/10 codes. Hospitals were stratified into low-, medium-, and high-volume tertiles based on annual institutional case volume of colorectal resection.

In order to examine overall morbidity, postoperative complications were analyzed both individually and as a composite of cerebrovascular (stroke), thromboembolic (deep vein thrombosis, pulmonary embolism), cardiac (cardiac arrest, myocardial infarction), respiratory (respiratory failure, prolonged mechanical ventilation, pneumonia), renal (acute kidney injury), and infectious (sepsis, abscess, wound infection) complications as well as hemorrhage using previously validated codes (S1 Table) [15]. Hospitalization costs were calculated from charges using hospital-specific cost-to-charge ratios and were inflation-adjusted to the 2020 Patient Health Care Index [16]. The primary outcome of interest was in-hospital mortality, while complications, costs, length of stay (LOS), and non-home discharge were also examined.

Categorical and continuous variables are reported as frequencies (%) and medians with interquartile range (IQR) and were compared using the Pearson’s chi-square and Mann-Whitney U tests, respectively. Multivariable mixed regression models were developed to evaluate the association of IDD with risk factors and outcomes of interest. Variable selection was performed by applying the Least Absolute Shrinkage and Selection Operator (LASSO), a regularization method that enhances model generalizability by minimizing overfitting and collinearity between independent variables [17]. The regression shrinkage incorporating adaptive penalty weights using LASSO has demonstrated high prediction accuracy with application to regression models by managing bias and variance trade-off in the selection of pertinent independent variables [18]. In order to examine whether the disparity in adverse events varied over time, interaction terms between year of admission and IDD were included in the multivariable logistic regression model to analyze risk-adjusted temporal trends. As previously described, the use of an interaction term computes the difference in predicted probabilities of adverse events between patients with and without IDD while holding at each consecutive year, ultimately calculating the difference in differences over the study period [19]. Regression results are reported as adjusted odds ratios (AOR) for dichotomous and beta coefficients (β) for continuous variables with 95% confidence intervals (95% CI). Statistical significance was set at α = 0.05. All statistical analyses were performed using Stata 16.1 (StataCorp, College Station, TX).

## Results

Among 722,736 patients who underwent resection for colorectal cancer, 2,846 (0.39%) had a diagnosis of IDD. Patients with IDD were younger compared to those without IDD (59 vs 68 years, p<0.001, Table 1). Furthermore, a greater proportion of IDD patients underwent an operation before the age of 45, when colorectal cancer screening is recommended for average-risk adults (15.3 vs 5.0%, p<0.001). The IDD cohort was less commonly female and more frequently publicly insured (Table 1). Compared to neurotypical patients, IDD patients had a higher Elixhauser Comorbidity Index (4 vs 3, p<0.001). Patients with IDD more commonly presented with bowel obstruction (14.4 vs 8.7%, p<0.001) and non-elective admission (46.8 vs 32.3%, p<0.001) and less commonly had minimally invasive operations (32.9 vs 40.5%, p<0.001, Table 1). In addition, IDD patients more frequently had an ostomy procedure (18.0 vs 14.3%, p = 0.01) and treatment at low-volume hospitals (44.6 vs 33.4%, p<0.001, Table 1). On sensitivity analysis, although rectal resection and total colectomy most frequently required an ostomy, only right colectomy showed statistically significant higher ostomy rates among adults with IDD (8.5 vs 5.1%, p = 0.01, S2 Table).

**Table 1:** Patient, operative and hospital characteristics among adults who underwent resection for colorectal cancer, stratified by diagnosis of intellectual or developmental disability (IDD). *IQR*: *Interquartile range*.

Parameter	No IDD (n = 719,890)	IDD (n = 2,846)	*p*-value
Age (years, median, IQR)	68 [58–78]	59 [51–67]	<0.001
Resection before age 45	5.0	15.3	<0.001
Female sex (%)	49.6	42.1	<0.001
*Race (%)*			0.01
White	74.6	75.6	
Black	11.2	14.2	
Hispanic	7.9	6.5	
Asian	3.3	1.6	
Other	3.0	2.1	
*Income Quartile (%)*			<0.001
Fourth (highest)	22.5	16.2	
Third	24.7	23.8	
Second	26.3	27.2	
First (lowest)	26.5	32.8	
*Payer Status (%)*			<0.001
Private	30.9	8.5	
Medicare	57.5	77.0	
Medicaid	7.0	13.8	
Uninsured	2.4	0.2	
Other	2.1	0.5	
*Comorbidities (%)*			
Elixhauser Comorbidity Index (median, IQR)	3 [2–5]	4 [3–5]	<0.001
Diabetes	23.9	18.0	<0.001
Hypertension	58.9	44.0	<0.001
Obesity	15.4	13.3	0.17
Coronary artery disease	14.5	5.4	<0.001
Chronic lung disease	14.7	11.6	0.04
Chronic liver disease	4.4	2.6	0.04
Presence of bowel obstruction (%)	8.7	14.4	<0.001
Non-elective admission (%)	32.3	46.8	<0.001
Minimally invasive approach (%)	40.5	32.9	<0.001
*Type of Resection (%)*			<0.001
Right colectomy	52.6	45.2	
Transverse colectomy	5.7	5.8	
Left colectomy	9.8	10.3	
Sigmoid colectomy	20.2	26.8	
Total colectomy	2.4	4.5	
Rectal resection	9.3	7.3	
Ostomy procedure (%)	14.3	18.0	0.01
*Hospital Operative Volume Status (%)*			<0.001
Low volume	33.4	44.6	
Medium volume	33.8	29.9	
High volume	32.7	25.5	
*Hospital Region (%)*			<0.001
Northeast	18.9	23.6	
Midwest	20.8	24.8	
South	40.3	38.1	
West	20.0	13.6	
*Hospital Teaching Status (%)*			0.004
Non-metropolitan	9.3	13.2	
Metropolitan non-teaching	27.8	28.2	
Metropolitan teaching	63.0	58.6	

Following multivariable risk adjustment for the factors tabulated in Table 2, IDD patients were of significantly younger age (AOR 0.90 [95% CI 0.89–0.91]) and had nearly 7-fold increased likelihood of resection before the age of 45 (6.85 [4.76–9.86], Table 2). In addition, patients in the IDD cohort had lower odds of being female (AOR 0.74 [95% CI 0.62–0.89], Table 2). IDD status was associated with a 1.4-fold increase in odds of non-elective admission (AOR 1.40 [95% CI 1.14–1.73], Table 2). In addition, IDD was associated with increased odds of undergoing sigmoid colectomy relative to right colectomy (AOR 1.37 [95% CI 1.11–1.71]), while there was no significant association between IDD and ostomy creation (Table 2). Compared to low-volume centers, medium- (AOR 0.76 [95% CI 0.61–0.95]) and high-volume (AOR 0.64 [95% CI 0.51–0.81]) centers were significantly less likely to treat IDD patients (Table 2).

**Table 2:** Patient, operative, and hospital characteristics associated with intellectual and developmental disability among patients undergoing resection for colorectal cancer. Model C-statistic: 0.88. *Ref*: *Reference*. *AOR*: *Adjusted odds ratio*. *CI*: *Confidence interval*.

Parameter	AOR [95% CI]	*p*-value
Age (per year)	0.90 [0.89–0.91]	<0.001
Resection before age 45	6.85 [4.76–9.86]	<0.001
Female sex (ref: male)	0.74 [0.62–0.89]	0.001
*Race*		
White	Ref	
Black	0.84 [0.64–1.09]	0.19
Hispanic	0.72 [0.51–1.03]	0.07
Asian	0.67 [0.35–1.28]	0.22
Other	0.52 [0.27–0.99]	0.05
*Payer Status*		
Private	Ref	
Medicare	30.8 [21.4–44.4]	<0.001
Medicaid	4.59 [3.15–6.69]	<0.001
Uninsured	0.19 [0.03–1.39]	0.10
Other	0.86 [0.26–2.82]	0.80
*Comorbidities*		
Elixhauser Comorbidity Index	1.05 [0.99–1.11]	0.12
Diabetes	0.80 [0.62–1.02]	0.07
Hypertension	0.76 [0.61–0.94]	0.01
Obesity	0.81 [0.61–1.08]	0.15
Coronary artery disease	0.46 [0.32–0.66]	<0.001
Presence of bowel obstruction	1.25 [0.96–1.64]	0.10
Non-elective admission	1.40 [1.14–1.73]	0.002
Minimally invasive approach	0.98 [0.81–1.19]	0.86
*Type of Resection*		
Right colectomy	Ref	
Transverse colectomy	1.18 [0.81–1.72]	0.39
Left colectomy	0.96 [0.70–1.30]	0.78
Sigmoid colectomy	1.37 [1.11–1.71]	0.004
Total colectomy	1.20 [0.76–1.89]	0.46
Rectal resection	0.96 [0.66–1.39]	0.83
Ostomy procedure	0.81 [0.63–1.04]	0.10
*Hospital Operative Volume Status*		
Low volume	Ref	
Medium volume	0.76 [0.61–0.95]	0.02
High volume	0.64 [0.51–0.81]	<0.001
*Hospital Region*		
Northeast	Ref	
Midwest	0.88 [0.68–1.15]	0.35
South	0.70 [0.53–0.89]	0.004
West	0.49 [0.36–0.67]	<0.001
*Hospital Teaching Status*		
Non-metropolitan	Ref	
Metropolitan non-teaching	0.90 [0.67–1.22]	0.51
Metropolitan teaching	0.88 [0.66–1.17]	0.39

Clinical and financial outcomes are shown in Table 3. Relative to neurotypical patients, IDD patients had significantly greater rates of in-hospital mortality (3.7 vs 1.9%, p = 0.003), perioperative complications (29.9 vs 21.0%, p<0.001) which were primarily respiratory (16.1 vs 7.6%, p<0.001) and infectious (11.4 vs 7.0%, p<0.001), as well as non-home discharge (39.8 vs 17.5%, p<0.001). Furthermore, the IDD cohort experienced significantly increased LOS (9 vs 6 days, p<0.001) and hospitalization costs ($23,500 vs $19,800, p<0.001). Following risk adjustment for the factors in Table 2, IDD status remained associated with over 2-fold greater odds of mortality (AOR 2.34 [95% CI 1.48–3.71]) and 1.4-fold increased odds of any complication (AOR 1.41 [95% CI 1.15–1.74]) particularly respiratory (Table 4). In addition, IDD was significantly associated with a +2.1-day increment in LOS (95% CI 1.5–2.7) and nearly 7-fold greater odds of non-home discharge (AOR 6.83 [95% CI 5.46–8.56], Table 4). Of note, adjusted rates of major adverse events, a composite of mortality and complications, remained greater among the IDD group over the study period with no significant trends over time (S1 Fig).

**Table 3:** Unadjusted clinical and financial outcomes stratified by diagnosis of intellectual or developmental disability (IDD) among patients undergoing colorectal cancer resection. *IQR*: *Interquartile range*.

Outcome	No IDD (n = 719,890)	IDD (n = 2,846)	*p*-value
In-hospital mortality (%)	1.9	3.7	0.003
*Complications (%)*			
Respiratory	7.6	16.1	<0.001
Infectious	7.0	11.4	<0.001
Cerebrovascular	0.4	0.7	0.23
Thromboembolic	1.4	1.6	0.78
Cardiac	1.5	1.2	0.58
Renal	10.1	7.7	0.06
Hemorrhagic	1.0	0.4	0.10
Any complication	21.0	29.9	<0.001
Length of stay (days, median, IQR)	6 [4–10]	9 [5–14]	<0.001
Cost ($1000s, median, IQR)	19.8 [13.9–29.6]	23.5 [16.0–35.6]	<0.001
Non-home discharge (%)	17.5	39.8	<0.001

**Table 4:** Adjusted outcomes associated with intellectual or developmental disability among patients undergoing colorectal cancer resection.

Outcome	AOR or ß [95% CI]	p-value
In-hospital mortality	2.34 [1.48–3.71]	<0.001
*Complications (%)*		
Respiratory	2.18 [1.70–2.80]	<0.001
Infectious	1.24 [0.93–1.66]	0.14
Cerebrovascular	2.31 [0.86–6.25]	0.10
Thromboembolic	0.85 [0.44–1.66]	0.64
Cardiac	0.88 [0.41–1.88]	0.74
Renal	0.67 [0.48–0.94]	0.02
Hemorrhagic	0.35 [0.09–1.40]	0.14
Any complication	1.41 [1.15–1.74]	0.001
Length of stay (days)	+2.13 [1.53–2.74]	<0.001
Cost ($)	+1400 [–400–3100]	0.12
Non-home discharge	6.83 [5.46–8.56]	<0.001

## Discussion

Using a nationally representative cohort of patients undergoing colorectal cancer resection, the present study examined the association of IDD with risk factors as well as in-hospital clinical and financial outcomes. Compared to patients without IDD, IDD patients underwent operations at a significantly younger age, were less likely to be treated at high-volume centers, and had increased odds of non-elective admission. Notably, patients with IDD faced significantly greater rates of mortality, postoperative complications, LOS, hospitalization costs, and non-home discharge relative to neurotypical patients. Several of these findings warrant further discussion.

We found that IDD patients were more likely to receive operations at ages below the screening threshold, suggesting the need for earlier colorectal cancer screening in the IDD population. Prior literature on IDD patients with cancer reported over a third (36%) of underlying causes of deaths to be gastrointestinal cancers, half of which were colorectal and anal cancers [20]. While our study cohort did not record a higher prevalence of obesity defined as BMI >30, previous studies have shown people with IDD have higher rates of sedentary behavior and obesity relative to the general population, both of which are recognized risk factors for colorectal cancer [3, 21]. Moreover, the IDD population is especially susceptible to social vulnerability factors that likely contribute to greater risk of developing colorectal cancer and receiving late diagnoses, including poverty, unemployment, social isolation, and housing insecurity [22–24]. These factors, in addition to increased physical and mental health comorbidities, cumulatively increase cancer risk in people with IDD and limit the timeliness and quality of oncologic care [6, 25]. Ensuring adequate communication between providers and IDD patients is crucial and often needs to be facilitated by surrogate healthcare decision-makers as well as trusted caregivers [26]. Individualized patient-centered care is warranted for this vulnerable population with consideration of the degree of IDD, BMI, functional status, caregiver support, and living conditions.

IDD patients were more frequently treated at low-volume hospitals, likely due to social barriers such as lower income and impaired access to experienced centers [18]. Historically built distrust of the healthcare system also plays a major role, as a systematic review of IDD patient experiences revealed an overarching fear of hospital encounters, institutional discrimination, delays in care, as well as reliance on caregivers for advocacy against negative stigma [27]. Of note, the inverse volume-outcome relationship in colorectal cancer surgery has been well-documented and is consistent with the inferior outcomes observed among those with IDD [28]. Our findings highlight a need for improved health policy and provider training regarding more equitable evaluation and care for patients with IDD [29]. Recent health delivery system innovations, such as the Developmental Disabilities Health Services’ Medical Home and University of Rhode Island’s Living Rite Centers, have streamlined care coordination to provide integrated primary care, mental health, and specialty medical care including neurology in the home and community settings [30]. Both models were associated with a reduction in ED visits and hospitalizations among IDD patients. Further integration of surgical care into these models and the creation of specialized, interdisciplinary centers of excellence may be crucial to improving access to care and outcomes among IDD patients with colorectal cancer. Moreover, health passports providing key information about how IDD individuals communicate, medical history, and support needs may help reduce barriers in access to high-quality care and were first introduced in the UK in 1990 and recently modified for use in the US in 2011 [31]. Legislation is crucial in the implementation of disability awareness training and supported decision making, as promoted by the United Nations Convention on the Rights of Persons with Disabilities [32]. Yet access to specialist health services and assistive devices for people with IDD is particularly low among low and middle income countries [33]. Global programs to improve access to affordable assistive technology, community-based models of healthcare delivery, and expansion of health coverage for IDD patients have been promoted by the World Health Organization to improve outcomes and mitigate catastrophic health expenditures [33].

IDD was significantly associated with non-elective admission, which may be a manifestation of delayed diagnosis and surgical treatment [34]. Individuals with IDD generally present at advanced stages of cancer [6, 7, 20]. A recent UK population-based study found that almost half (45%) of cancers among IDD patients were diagnosed at stage IV [20]. Variations in abilities to communicate among patients with IDD may also cause providers to overlook warning symptoms, thus delaying diagnosis and allowing cancer to progress without treatment [35, 36]. Notably, people with IDD have been shown to have significantly lower likelihood of undergoing preventive health screenings [37]. Adherence to screening guidelines may be challenging due to lack of accessible information and poor understanding of the screening process. Providing visual guides with images of how to complete fecal occult blood tests may particularly benefit individuals with IDD [8]. Interventions such as modified pain assessment tools, adaptive communication devices, transportation services, and additional support with cancer navigators should be made accessible to IDD patients, caregivers and providers [36–39]. Practical strategies to improve oncologic care for people with IDD include increasing clinic visit time and incorporating level of ability, overall health, and goals of care into advanced care directives and routine documentation by providers [7].

Respiratory complications were significantly increased among IDD patients compared to neurotypical patients. An estimated 15% of adults with IDD experience difficulty with eating, drinking, and swallowing [40]. These patients are at greater risk of aspiration and pulmonary infection during mealtime, which may be particularly exacerbated after gastrointestinal surgery. As IDD conditions such as cerebral palsy show increasing survival rates into adulthood, adult pulmonologists and critical care providers often lack experience with this patient population [41]. Preoperative evaluation of swallow function and adjusting perioperative diet appropriately with mealtime support is warranted to decrease risk of postoperative respiratory complications. In addition, patients with severe IDD are known to have higher baseline end-tidal CO_2_ and increased risk of respiratory illness due to kyphoscoliosis and increased psychiatric medication use [42]. Knowing baseline end-tidal CO_2_ and oximetry data may help guide management with individualized perioperative care plans. Implementing postoperative protocols such as prolonged respiratory monitoring and care coordinator consults may also be beneficial for patients with IDD [11].

The present study has several limitations due to its retrospective nature and use of administrative data. The NIS does not capture outpatient information such as colorectal cancer screening or preoperative evaluation. In addition, granular clinical data including time of cancer diagnosis, cancer staging, and anatomic or physiologic complexity of each procedure were unable to be assessed, which may be confounding factors. Earlier time to diagnosis would allow for a more comprehensive cancer care plan including neoadjuvant therapy if necessary, which may increase feasibility of resection and explain the improved outcomes among patients without IDD [43]. In addition, individuals with IDD generally present at more advanced stages of cancer, which may underlie the increased rate of mortality and complications [6, 7, 20]. Further investigation is necessary to identify modifiable risk factors and determine whether inadequate preoperative screening and evaluation or institutional discrimination regardless of staging or time of diagnosis are greater contributors to adverse outcomes among IDD patients. The clinical and financial endpoints analyzed were limited to the duration of admission and long-term outcomes such as readmissions and reoperations were not available. Admission from non-home facility was not captured in the NIS, thus potentially overestimating rates of non-home discharge. Furthermore, the IDD cohort included adults with varying levels of IDD severity, which may impact the risk for adverse outcomes [44]. ICD coding is influenced by provider and center practices among participating hospitals in the NIS, and the transition from ICD-9 to ICD-10 may also introduce variations in coding. In particular, the observed prevalence of obesity was much lower than prior reported estimates at 30% in the IDD population, suggesting underutilization of BMI coding [21]. Due to the low prevalence of IDD, our study cohort was relatively small in sample size. Nevertheless, the proportion of colorectal cancer resection patients with IDD was similar to the proportion previously reported in other surgical studies [11, 12]. Despite these limitations, we utilized the largest all-payer inpatient database and robust statistical methods to enhance the generalizability of our findings at the national level.

### Conclusions

The present study used a nationally representative database to demonstrate that IDD was associated with younger age, non-elective admission, and treatment at lower-volume centers among patients undergoing colorectal cancer resection. Furthermore, patients with IDD experienced significantly increased mortality, complications, and resource use relative to neurotypical patients. The disproportionate burden of adverse clinical and financial outcomes highlights a particularly vulnerable population and emphasizes the critical need to improve colorectal cancer care for individuals with IDD. Given the persistent disparity in major adverse events over the past decade, systematic approaches to eliminate disparities in colorectal cancer screening and optimize surgical outcomes among patients with IDD are warranted.

## Supporting information

S1 Fig Temporal trends in risk-adjusted rates of major adverse events (MAE) stratified by diagnosis of intellectual or developmental disability (IDD). MAE was defined as a composite of in-hospital mortality and complications.
(DOCX)

S1 Table Administrative international classification of diseases, 9th and 10th revision (ICD-9/10) diagnosis and procedure codes for patients with intellectual or developmental disability undergoing colorectal cancer resection. (DOCX)

S2 Table Sensitivity analysis of ostomy rates by type of colorectal cancer resection among patients with intellectual or developmental disability (IDD). (DOCX)

## References

[1] Anderson LL, Larson SA, MapelLentz S, et al. A systematic review of US studies on the prevalence of intellectual or developmental disabilities since 2000. Intellect Dev Disabil. 2019;57:421–438. 31568738 10.1352/1934-9556-57.5.421

[2] Patja K, Eero P, Iivanainen M. Cancer incidence among people with intellectual disability. J Intellect Disabil Res. 2001;45:300–307. doi: 10.1046/j.1365-2788.2001.00322.x 11489051

[3] Willis D, Samalin E, Satgé D. Colorectal cancer in people with intellectual disabilities. Oncology. 2018;95(6):323–36. doi: 10.1159/000492077 30173217

[4] Cooper SA, McLean G, Guthrie B, et al. Multiple physical and mental health comorbidity in adults with intellectual disabilities: population-based cross-sectional analysis. BMC Fam Pract. 2015;16:110. doi: 10.1186/s12875-015-0329-3 26310664 PMC4551707

[5] Raouafi S, Achiche S, Raison M. Socioeconomic disparities and difficulties to access to healthcare services among Canadian children with neurodevelopmental disorders and disabilities. Epidemiol Health. 2018;40:e2018010. doi: 10.4178/epih.e2018010 29642656 PMC6004430

[6] Krahn GL, Fox MH. Health disparities of adults with intellectual disabilities: what do we know? what do we do? J Appl Res Intellect Disabil. 2014;27:431–446. doi: 10.1111/jar.12067 23913632 PMC4475843

[7] McMahon M, McCallion P, McCarron M. An invisible population: Late‐stage cancer diagnosis for people with intellectual or developmental disability. Cancer. 2023. doi: 10.1002/cncr.35132 38014927

[8] Satgé D, Habib-Hadef S, Otandault A, Samalin E, Trétarre B. Advocacy for colorectal cancer screening and awareness in people with intellectual disability. Journal of Gastrointestinal Oncology. 2023;14(3):1650. doi: 10.21037/jgo-22-998 37435211 PMC10331771

[9] Cairns AL, Schlottmann F, Strassle PD, Di Corpo M, Patti MG. Racial and socioeconomic disparities in the surgical management and outcomes of patients with colorectal carcinoma. World journal of surgery. 2019;43:1342–50. doi: 10.1007/s00268-018-04898-5 30610271

[10] Le H, Ziogas A, Lipkin SM, Zell JA. Effects of socioeconomic status and treatment disparities in colorectal cancer survival. Cancer Epidemiology Biomarkers & Prevention. 2008;17(8):1950–62. doi: 10.1158/1055-9965.EPI-07-2774 18708384

[11] Zondlak AN, Oh EJ, Neiman PU, Fan Z, Taylor KK, Sangji NF, et al. Association of Intellectual Disability with Delayed Presentation and Worse Outcomes in Emergency General Surgery. Annals of Surgery. 2023;278(5):e1118–22. doi: 10.1097/SLA.0000000000005863 36994738

[12] Hand BN, Hyer JM, Schenk A, Coyne A, Gilmore D, Wang L, et al. Comparing kidney transplant rates and outcomes among adults with and without intellectual and developmental disabilities. JAMA surgery. 2023;158(4):386–92. doi: 10.1001/jamasurg.2022.7753 36790769 PMC9932938

[13] HCUP National Inpatient Sample (NIS). Healthcare Cost and Utilization Project (HCUP), Agency for Healthcare Research and Quality, Rockville, MD. 2012 (accessed Jan 18 2024). Retrieved from www.hcup-us.ahrq.gov/nisoverview.jsp.

[14] Elixhauser A, Steiner C, Harris DR, Coffey RM. Comorbidity measures for use with administrative data. Med Care. 1998;36:8–27, doi: 10.1097/00005650-199801000-00004 9431328

[15] Storesund A, Haugen AS, Hjortås M, et al. Accuracy of surgical complication rate estimation using ICD-10 codes. Journal of British Surgery. 2019;106(3):236–44. doi: 10.1002/bjs.10985 30229870 PMC6519147

[16] Medical Expenditure Panel Survey. Using appropriate price indices for expenditure comparisons. Agency for Healthcare Research and Quality. 2021 (accessed Jan 18 2024). Retrieved from https://meps.ahrq.gov/about_meps/Price_Index.shtml

[17] Tibshirani R. Regression shrinkage and selection via the lasso. J R Stat Soc Ser B. 1996;58:267–288.

[18] Daneshvar A, Mousa G. Regression shrinkage and selection via least quantile shrinkage and selection operator. PLoS One. 2023;18(2):e0266267. doi: 10.1371/journal.pone.0266267 36795659 PMC9934385

[19] Karaca‐Mandic P, Norton EC, Dowd B. Interaction terms in nonlinear models. Health services research. 2012;47(1pt1):255–74. doi: 10.1111/j.1475-6773.2011.01314.x 22091735 PMC3447245

[20] Heslop P, Cook A, Sullivan B, Calkin R, Pollard J, Byrne V. Cancer in deceased adults with intellectual disabilities: English population-based study using linked data from three sources. BMJ open. 2022;12(3). doi: 10.1136/bmjopen-2021-056974 35332044 PMC8948391

[21] Rimmer JH, Yamaki K: Obesity and intellectual disability. Ment Retard Dev Disabil Res Rev. 2006; 12: 22–27. doi: 10.1002/mrdd.20091 16435329

[22] Emerson E, Graham H, Hatton C. The measurement of poverty and socioeconomic position in research involving people with intellectual disability. Int Rev Res Ment Retard. 2006;32:77‐108.

[23] McMahon M, Bowring DL, Hatton C. Not such an ordinary life: a comparison of employment, marital status and housing profiles of adults with and without intellectual disabilities. Learn Disabil Rev. 2019;24(4):213‐221. doi: 10.1108/tldr‐03‐2019‐0014

[24] Wormald AD, McCallion P, McCarron M. The antecedents of loneliness in older people with an intellectual disability. Res Dev Disabil. 2019;85:116‐130. doi: 10.1016/j.ridd.2018.11.009 30551091

[25] McMahon M, Hatton C. A comparison of the prevalence of health problems among adults with and without intellectual disability: a total administrative population study. J Appl Res Intellect Disabil. 2021;34(1):316‐325. doi: 10.1111/jar.12785 32734651

[26] Boonman AJ, Cuypers M, Leusink GL, Naaldenberg J, Bloemendal HJ. Cancer treatment and decision making in individuals with intellectual disabilities: a scoping literature review. The Lancet Oncology. 2022;23(4):e174–83. doi: 10.1016/S1470-2045(21)00694-X 35358466

[27] Iacono T, Bigby C, Unsworth C, et al. A systematic review of hospital experiences of people with intellectual disability. BMC Health Serv Res. 2014;14:505. doi: 10.1186/s12913-014-0505-5 25344333 PMC4210514

[28] Huo YR, Phan K, Morris DL, Liauw W. Systematic review and a meta-analysis of hospital and surgeon volume/outcome relationships in colorectal cancer surgery. Journal of gastrointestinal oncology. 2017;8(3):534. doi: 10.21037/jgo.2017.01.25 28736640 PMC5506277

[29] Lennox NG, Diggens J, Ugoni A. Health care for people with an intellectual disability: General Practitioners’ attitudes, and provision of care. J Intellect Dev Disabil. 2000;25:127–133.

[30] Ruiz S, Giuriceo K, Caldwell J, Snyder LP, Putnam M. Care coordination models improve quality of care for adults aging with intellectual and developmental disabilities. Journal of Disability Policy Studies. 2020;30(4):191–201.

[31] Heifetz M, Lunsky Y. Implementation and evaluation of health passport communication tools in emergency departments. Research in developmental disabilities. 2018;72:23–32. doi: 10.1016/j.ridd.2017.10.010 29080483

[32] Sherlaw W, Hudebine H. The United Nations Convention on the rights of persons with disabilities: Opportunities and tensions within the social inclusion and participation of persons with disabilities. Alter. 2015;9(1):9–21.

[33] Bright T, Wallace S, Kuper H. A systematic review of access to rehabilitation for people with disabilities in low-and middle-income countries. International journal of environmental research and public health. 2018;15(10):2165. doi: 10.3390/ijerph15102165 30279358 PMC6210163

[34] Howard R, Hendren S, Patel M, Gunaseelan V, Wixson M, Waljee J, et al. Racial and Ethnic Differences in Elective Versus Emergency Surgery for Colorectal Cancer. Annals of Surgery. 2023;278(1):e51–7. doi: 10.1097/SLA.0000000000005667 35950753 PMC11062257

[35] Lewis MA, Lewis CE, Leake B, et al. The quality of health care for adults with developmental disabilities. Public Health Rep. 2002;117:174–184. doi: 10.1093/phr/117.2.174 12357002 PMC1497422

[36] Ely E, Chen-Lim ML, Carpenter KM, et al. Pain assessment of children with autism spectrum disorders. J Dev Behav Pediatr JDBP. 2016;37:53–61. doi: 10.1097/DBP.0000000000000240 26703326

[37] Ouellette-Kuntz H, Coo H, Cobigo V, et al. Uptake of colorectal cancer screening among Ontarians with intellectual and developmental disabilities. PLoS One 2015;10:e0118023. doi: 10.1371/journal.pone.0118023 25689849 PMC4331499

[38] Chaudhary K, Bagharwal P, Wadhawan S. Anesthesia for intellectually disabled. J Anaesthesiol Clin Pharmacol. 2017;33:432–440. doi: 10.4103/joacp.JOACP_357_15 29416231 PMC5791252

[39] Stallard P, Williams L, Velleman R, et al. The development and evaluation of the pain indicator for communicatively impaired children (PICIC). Pain. 2002;98:145–149. doi: 10.1016/s0304-3959(02)00038-6 12098626

[40] Perez CM, Wagner AP, Ball SL, White SR, Clare IC, Holland AJ, et al. Prognostic models for identifying adults with intellectual disabilities and mealtime support needs who are at greatest risk of respiratory infection and emergency hospitalisation. Journal of Intellectual Disability Research. 2017;61(8):737–54. doi: 10.1111/jir.12376 28497469 PMC5518212

[41] Desai B, Lesser DJ, Sunwoo BY. Managing Pulmonary Complications in Adults with Cerebral Palsy-A Vulnerable Transition. CHEST Pulmonary. 2023;1(3):100025. 10.1016/j.chpulm.2023.100025 PMC13420473 42548418

[42] Jacob R, Nelkenbaum A, Merrick J, Brik R. Capnography in patients with severe neurological impairment. Research in developmental disabilities. 2014;35(6):1259–63. doi: 10.1016/j.ridd.2014.03.015 24685942

[43] Buccafusca G, Proserpio I, Tralongo AC, Giuliano SR, Tralongo P. Early colorectal cancer: diagnosis, treatment and survivorship care. Critical reviews in oncology/hematology. 2019;136:20–30. doi: 10.1016/j.critrevonc.2019.01.023 30878125

[44] Heslop P, Lauer E, Hoghton M. Mortality in people with intellectual disabilities. Journal of Applied Research in Intellectual Disabilities. 2015;28(5):367–72. doi: 10.1111/jar.12196 26011045

