# Supplementary material for: Disparities in outcomes of colorectal cancer surgery among adults with intellectual and developmental disabilities
Source: PLoS One. 2024 Aug 27;19(8):e0308938. doi: 10.1371/journal.pone.0308938 (PMC11349222; doi:10.1371/journal.pone.0308938)

Supplemental Figure 1. Temporal trends in risk-adjusted rates of major adverse events (MAE) stratified by diagnosis of intellectual or developmental disability (IDD). MAE was defined as a composite of in-hospital mortality and complications.


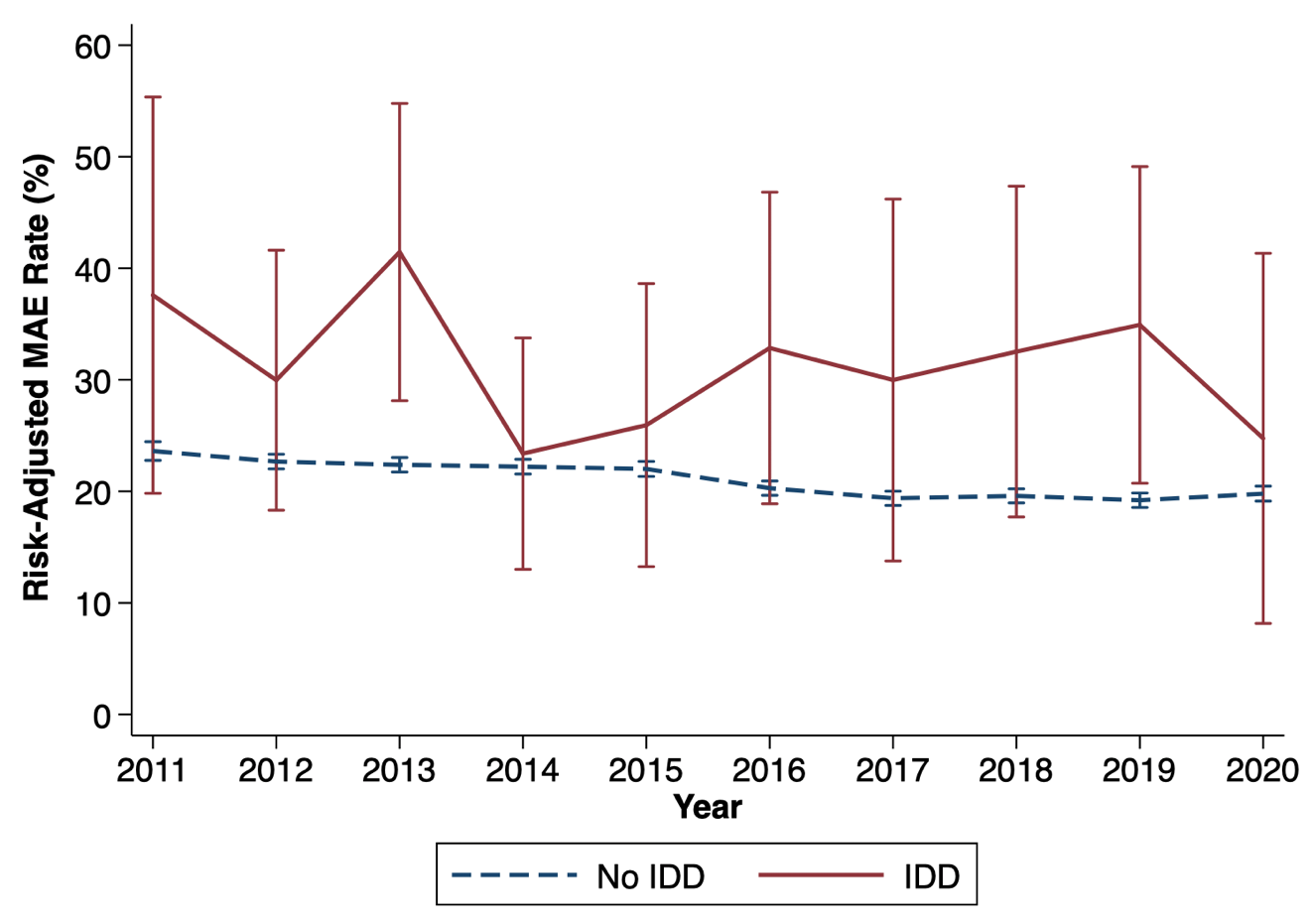

Supplement: S1 Fig — MAE was defined as a composite of in-hospital mortality and complications. (DOCX) [file pone.0308938.s001.docx]
