# Supplementary material for: Disparities in outcomes of colorectal cancer surgery among adults with intellectual and developmental disabilities
Source: PLoS One. 2024 Aug 27;19(8):e0308938. doi: 10.1371/journal.pone.0308938 (PMC11349222; doi:10.1371/journal.pone.0308938)
Supplement: S1 Table — (DOCX) [file pone.0308938.s002.docx]

Supplemental Table 1. Administrative International Classification of Diseases, 9th and 10th Revision (ICD-9/10) diagnosis and procedure codes for patients with intellectual or developmental disability undergoing colorectal cancer resection.

|  | **ICD-9** | **ICD-10** |
| --- | --- | --- |
| **Intellectual disability** | 317, 318, 319 | F70, F71, F72, F73, F78, F79 |
| Pervasive developmental disorders | 299 | F84 |
| Cerebral palsy | 343 | G80 |
| Down syndrome | 758.0 | Q90 |
|  |  |  |
| **Colectomy** |  |  |
| Right colectomy |  |  |
| Open | 45.73 | 0DBF0ZZ, 0DBK0ZZ, 0DTF0ZZ, 0DTK0ZZ |
| Laparoscopic | 17.33 | 0DBF4ZZ, 0DBK4ZZ, 0DTF4ZZ, 0DTK4ZZ |
| Left colectomy |  |  |
| Open | 45.75 | 0DBG0ZZ, 0DBM0ZZ, 0DTG0ZZ, 0DTM0ZZ |
| Laparoscopic | 17.35 | 0DBG4ZZ, 0DBM4ZZ, 0DTG4ZZ, 0DTM4ZZ |
| Transverse colectomy |  |  |
| Open | 45.74 | 0DBL0ZZ, 0DTL0ZZ |
| Laparoscopic | 17.34 | 0DBL4ZZ, 0DTL4ZZ |
| Sigmoid colectomy |  |  |
| Open | 45.76 | 0DBN0ZZ, 0DTN0ZZ |
| Laparoscopic | 17.36 | 0DBN4ZZ, 0DTN4ZZ |
| Total colectomy |  |  |
| Open | 45.71, 45.82 | 0DBE0ZZ, 0DTE0ZZ |
| Laparoscopic | 17.31, 45.81 | 0DBE4ZZ, 0DTE4ZZ |
| **Rectal Resection** |  |  |
| Open | 48.51, 48.52, 48.62 | 0DTP0ZZ, 0DBP0ZZ |
| Laparoscopic | 48.51 | 0DTP4ZZ, 0DBP4ZZ |
| **Robot-Assisted** | 17.42, 17.44, and laparoscopic procedure code | 8E0W4CZ, 8E0W8CZ, and laparoscopic procedure code |
| **Colorectal cancer** | 153, 154 | C18, C19, C20 |
|  |  |  |
| **Comorbidities** |  |  |
| Diabetes | 250 | E10, E11, E13 |
| Hypertension | 401, 402, 403, 404, 405 | I10, I11, I12, I13, I15 |
| Obesity | E66, Z68.3, Z68.4 | 278.0, V85.3, V85.4 |
| Coronary artery disease | 411, 412, 413, 414 | I20, I24, I25 |
| Chronic pulmonary disease | 416.8, 416.9, 506.4, 508.8, 500, 501, 502, 503, 504, 505, 490, 491, 492, 493, 494, 495, 496 | I27.8, I27.9, J68.4, J70.1, J70.3, J40, J41, J42, J43, J44, J45, J46, J47, J60, J61, J62, J63, J64, J65, J66, J67 |
| Chronic liver disease | 070.22, 070.23, 070.32, 070.33, 070.44, 070.54, 070.6, 070.9, 456.0, 456.1, 456.2, 572.2, 572.3, 572.4, 572,8, 573.3, 573.4, 573.8, 573.9, V42.7, 570, 571 | K70, K72, K73, K74, B18, I85, K76.3, K76.4, K76.5, K76.6, K76.7, K76.8, K76.9, Z94.4, K71.1, K71.3, K71.4, K71.5, K71.7, K76.0, K76.2, I86.4, I98.2 |
|  |  |  |
| Bowel obstruction | 560.89, 560.9 | K56.6 |
| **Stoma Creation** |  |  |
| Colostomy | 46.03, 46.10, 46.11, 46.13, 46.14, 48.62 | 0D1K0Z4, 0D1K4Z4, 0D1K8Z4, 0D1L0Z4, 0D1L4Z4, 0D1L8Z4, 0D1N0Z4, 0D1N4Z4 |
| Ileostomy | 46.01, 46.20, 46.21, 46.22, 46.23 | 0D190Z4, 0D194Z4, 0D198Z4, 0D1A0Z4, 0D1A4Z4, 0D1A8Z4, 0D1B8Z4, 0D1B0Z4, 0D1B4Z4, 0D1B8Z4 |
|  |  |  |
| **Complications** |  |  |
| Stroke | 433.01, 433.11, 433.21, 433.31, 433.81, 433.91, 434.01, 434.11, 434.91, 437.0, 437.1, 437.4, 437.5, 437.7, 437.9, 997.01, 997.02, 431, 432.0, 432.1, 432.9, 430 | I63, I67.2, I67.81, I67.82, I67.89, I67.7, I67.5, I67.9, G97.81, G97.82, I97.811, I97.821, I61.9, I62.1, I60.9, I62.9, I62.0, I62.1 |
| Deep Vein Thrombosis | 451.1, 451.2, 451.81, 451.9, 453.2, 453.40, 453.41, 453.42, 453.8, 453.9 | I82.220, I82.4, I82.6, I82.A1, I82.B1, I82.C1, I82.290, I82.890, I82.91, I80.9, I80.3 |
| Pulmonary Embolism | 415.1 | I26 |
| Cardiac Arrest | 427.5 | I46.2, I46.8, I46.9 |
| Myocardial infarction | 410 | I21 |
| Respiratory Failure | 518.81, 518.51, 518.53, 518.84 | J96.00, J96.90, J96.20, J95.821, J95.822 |
| Prolonged Mechanical Ventilation | 96.72 | 5A1955Z |
| Pneumonia | 480, 481, 482, 483, 485, 486, 997.31, 997.32 | J12, J13, J14, J15, J16, J18, J95.851, J95.89 |
| Acute Kidney Injury | 584 | N17 |
| Sepsis | 038, 995.91, 995.92, 999.3, 998.51, 998.59 | A40, A41, R65.20, T814XXA, K68.11 |
| Abscess | 569.5, 790.7 | R78.81, K63.0 |
| Wound infection | 998.31, 998.32, 998.5 | T81.32XA, T81.31XA, T81.4XXA, K68.11 |
| Hemorrhage | 998.11 | D78.01, D78.02, D78.21, D78.22, E36.01, E36.02, E89.810, E89.811, G97.31, G97.32, G97.51, G97.52, H59.111, H59.112, H59.113, H59.119, H59.121, H59.122, H59.123, H59.129, H59.311, H59.312, H59.313, H59.319, H59.321, H59.322, H59.323, H59.329, H95.21, H95.22, H95.41, H95.42, I97.410, I97.411, I97.418, I97.42, I97.610, I97.611, I97.618, I96.620, J95.61, J95.62, J95.830, J95.831, K91.61, K91.62, K91.840, K91.841, L76.01, L76.02, L76.21, L76.22, M96.810, M96.811, M96.830, M96.831, N99.61, N99.62, N99.820, N99.821 |
