# Supplementary material for: Disparities in outcomes of colorectal cancer surgery among adults with intellectual and developmental disabilities
Source: PLoS One. 2024 Aug 27;19(8):e0308938. doi: 10.1371/journal.pone.0308938 (PMC11349222; doi:10.1371/journal.pone.0308938)
Supplement: S2 Table — (DOCX) [file pone.0308938.s003.docx]

Supplemental Table 2. Sensitivity analysis of ostomy rates by type of colorectal cancer resection among patients with intellectual or developmental disability (IDD).

| **Type of Resection (%)** | **No IDD** | **IDD** | **p-value** |
| --- | --- | --- | --- |
| Right colectomy | 5.1 | 8.5 | 0.01 |
| Transverse colectomy | 11.6 | 9.1 | 0.66 |
| Left colectomy | 18.3 | 25.5 | 0.15 |
| Sigmoid colectomy | 18.6 | 21.6 | 0.35 |
| Total colectomy | 40.9 | 45.9 | 0.61 |
| Rectal resection | 47.9 | 42.4 | 0.48 |
